# Trends and disparities in sleep quality and duration in older adults in China from 2008 to 2018: A national observational study

**DOI:** 10.3389/fpubh.2023.998699

**Published:** 2023-02-17

**Authors:** Zihao Tao, Yuting Feng, Jue Liu, Liyuan Tao

**Affiliations:** ^1^Research Center of Clinical Epidemiology, Peking University Third Hospital, Beijing, China; ^2^School of Basic Medical Sciences, Peking University, Beijing, China; ^3^School of Traditional Chinese Medicine, Beijing University of Chinese Medicine, Beijing, China; ^4^School of Public Health, Peking University, Beijing, China; ^5^Medical Examination Centre, Peking University Third Hospital, Beijing, China

**Keywords:** sleep quality, sleep duration, older adult, trend, disparities

## Abstract

**Background:**

Poor sleep status as a common concern is a risk factor for many health problems among older people. China with an aging society lacks relevant nationwide data on the sleep status among older people. Therefore, the purpose of this study was to investigate trends and disparities in sleep quality and duration among older adults, and exploring influencing factors of poor sleep in China between 2008 and 2018.

**Method:**

We used the four-waves data of the Chinese Longitudinal Healthy Longevity Survey (CLHLS) from 2008 to 2018. Sleep quality and average sleep hours per day was investigated by using questionnaires in the CLHLS. We categorized sleep duration as three groups including ≤5 h (short duration), 5–9 h (normal duration), or ≥9 h (long duration) per day. Multivariate logistic regression models were used to examine trends and risk factors of poor sleep quality, short sleep duration, and long sleep duration.

**Results:**

The prevalence of poor sleep quality significantly increased from 34.87% in 2008 to 47.67% in 2018 (*p* < 0.05). Short sleep duration significantly increased from 5.29 to 8.37%, whereas long sleep duration decreased from 28.77 to 19.27%. Multivariate analysis showed that female sex, poor economic status, a greater number of chronic diseases, underweight, poor self-reported quality of life, and poor self-reported health were associated with poor sleep quality and short sleep duration (*p* < 0.05).

**Conclusion:**

Our findings revealed that older adults had increased prevalence of poor sleep quality and short sleep duration from 2008 to 2018. More attention should be paid to the increased sleep problems among older adults and early interventions should be made to improve sleep quality and guarantee enough sleep time.

## Introduction

Sleep quality and duration are important health topics. Unfortunately, epidemiological studies have shown that sleep problems are very common among older people. Previous studies showed that the prevalence of sleep problems was 16.6, 28.9, and 31.2% in Denmark, Japan and Poland, respectively (1, 2). Some studies had reported increased trends of sleep problems because of the increasing physical and psychological issues in facing of the rapidly changing world (3–5). China as the country with the largest number of older people in the world, has the increasing trend of aging which means the burden on families and public health care (6). According to the National Bureau of Statistics of China, there were 109.56 million people over the age of 65 years in China in 2008, accounting for 8.25% of the total population (7). By 2018, this number had increased to 167.24 million, accounting for 11.90% (7). Although several urban and regional studies reported that the high prevalence of poor sleep quality among older people ranged from 33.8 to 49.7% (8–11), there was a lack of studies on long-term trends in sleep status in China.

Many studies had showed that sleep problems were associated with the increased risk of adverse outcomes among older people, thus sleep should be paid attention and surveilled. Evidence reported that poor sleep quality increased the risks of fall (2), physical disability (12), and hypertension among older people (13). A U-shaped dose–response relationship existed in the relationship between sleep duration and other health problems, such as cognitive function decline (14), osteoporosis (15), type 2 diabetes (16), and coronary heart disease (17), even mortality (18). Maintaining good sleep quality and normal sleep duration is very important for the health among older people. Therefore, considering the above information on the common sleep problems and its harm among older people, the identification of related risk factors is of great importance for public health and clinically to develop effective interventions of sleep problems.

Previous studies have shown that some demographic factors, socioeconomic status, lifestyle habits, and health conditions played crucial roles in sleep quality and duration among older people (14, 18–22). Understanding the characteristics of the demographic factors, socioeconomic status and health conditions was useful to identify the risk population. For example, previous studies reported that female (22), people with higher educational level (19) and increased number of chronic diseases (21) may be poor sleepers. Meanwhile, exploring the association between lifestyle habits and sleep was benefit for improving sleep by establishing good lifestyle habits or decreasing bad lifestyle habits (20). However, compared with demographic factors, socioeconomic status and health conditions, the lifestyle habits, especially some detail activities, were investigated rarely among older people, such as housework, having pets or gardening, reading books, playing cards, watching television, and social participation.

Based on the above background on lacked national research evidence about long-term change trends of sleep and related influencing factors among the Chinese older people, the purpose of this study was to investigate trends and disparities in the quality of sleep and sleep duration among adults aged 65 years and older from 2008 to 2018 using the Chinese Longitudinal Healthy Longevity Survey (CLHLS) data.

## Methods

### Study population and data source

This was a national observational study using the CLHLS data from 2008 and 2018. The CLHLS as the first national longitudinal survey of older people in a developing country, was launched in 1998, accounting for about 90% of the country's population from a randomly selected half of the counties and cities in 23 of 31 provinces in China. A targeted random-sample design was adopted to ensure representativeness. All of the centenarians of the sampled counties and cities agreed voluntarily to participate in the study. This study was established in 1998, with subsequent follow-up and recruitment of new participants in 2000, 2002, 2005, 2008, 2011, 2014, and 2018. The collected data includes demographic characteristics, family and residential characteristics, marital status, living arrangements, social and economic characteristics, health, and other individual data for a large number of older individuals (23). To account for deaths and people who lost follow-up, CLHLS enrolled new participants according to similar sex, age, and other characteristics of the missing persons to ensure consistency of the study. To ensure the quality of the survey, the project team had strictly and carefully trained investigators to conduct the household surveys to ensure the quality of the survey. All the surveys were face-to-face interviews conducted at the participant's home. Each participant provided a signed informed consent form; this was signed by the next of kin if the participant could not sign it. More details about the study design of the CLHLS can be found elsewhere (23).

There was a total of 50,870 participants in the surveys during 2008–2018 (16,954 in 2008, 10,850 in 2011, 7,192 in 2014, and 15,874 in 2018). We excluded 2,759 participants who had missing data on sleep quality and 740 participants aged below 65 years, yielding 47,371 participants (93.12%) in the final study population.

### Assessment of sleep status

Sleep quality and sleep duration were assessed by using two questions: “How is your sleep quality now?” and “How many hours do you sleep on average now?”, respectively, which were both commonly used in previous studies (14, 24, 25). The response options of sleep quality included five categories: excellent, good, average, not good, and very bad, we defined poor sleep quality as a response of average/bad/very bad based on the research from Gu et al., others were good sleep quality (25). The 5 categories (excellent, good, average, not good, and very bad) were binary divided as poor sleep quality (encompassing average, not good, and very bad subcategories) and good sleep quality (encompassing excellent and good subcategories) (25). We divided sleep duration for adults over age 65 years into three groups, according to the classification on sleep duration (<5 h as short sleep duration, 5–9 h as normal, and more than 9 h as long sleep duration) from National Sleep Foundation (26) and the research from Gu et al. (25). In the sensitivity analysis, we further divided the participants into three groups (<7, 7–9, >9 h) for more categories.

### Assessment of related factors

Based on previous research (14, 24, 25), we included relevant influencing factors of sleep status, including demographic factors, socioeconomic status, lifestyle habits, and health conditions. Demographic factors included investigation year, sex (male or female), age group (65–79, 80–89, 90–99, and ≥100 years), marital status (unmarried, married, or divorced or widowed), and residence (urban or rural).

Socioeconomic status included economic status compared with other local people (wealthy, average, or poor), living arrangements (living with family members, living in an institution, or living alone), and years of schooling (0 or ≥1 year).

Lifestyle habits included smoking status (never, previous, or current), alcohol intake (never, previous, or current), regular exercise (never, previous, or current), dietary diversity score (poor, moderate, or good), housework (nearly every day, sometimes, or never), outdoor activities (nearly every day, sometimes, or never), having pets or gardening (nearly every day, sometimes, or never), reading books (nearly every day, sometimes, or never), raising poultry (nearly every day, sometimes, or never), playing cards (nearly every day, sometimes, or never), watching television (TV; nearly every day, sometimes, or never), and social participation (nearly every day, sometimes, or never).

Health conditions included body mass index (BMI; underweight, normal weight, overweight, or obesity), number of chronic diseases (0, 1, or ≥2), activities of daily living (ADL; independent or disabled), self-reported quality of life (good, average, or poor) and self-reported health (good, average, or poor), and cognitive impairment (yes, no). The height and weight of participants were measured by calibrated instruments, as described previously (27). BMI was categorized as underweight (<18.5 kg/m^2^), normal weight (18.5–24.9 kg/m^2^), overweight (25–29.9 kg/m^2^), and obese (≥30 kg/m^2^), according to the cutoff values suggested by the World Health Organization. As for dietary diversity, consumption frequencies of nine food groups (meat, vegetables, fish, eggs, fruits, legumes, milk, tea, and nuts) were recorded, and the dietary diversity score (0–9) was calculated and categorized according to the recommendations by the Food and Agriculture Organization of the United Nations and previous research (28, 29). ADL refers to basic personal care tasks of everyday life. In this study, ADL in disability was defined as self-reported difficulty with any of the following ADL items: dressing, eating, bathing, continence, toileting, cleaning, and indoor movement (24, 30). In compliance with the previous studies (31, 32), cognitive function was measured by t cognitive assessment tool, the Chinese version of the Mini-Mental State Examination (MMSE) questionnaire, which consists of 11 questions covering orientation, registration, attention, calculation, recall, and language abilities with a total score of 30, which had shown good validity and reliability (33, 34). CLHLS participants who scored <18 were classified as having cognitive impairment, whereas participants with a score of 18 or higher were classified as having no cognitive impairment (31, 32).

### Data analysis

Baseline characteristics were described as percentages for categorical variables and median (interquartile range [IQR]) for continuous variables. We tested statistical differences using the chi-square test for categorical variables and the *t*-test for normally distributed continuous variables according to sleep quality and sleep duration. We used multivariate logistic regression models to analyze risk factors related to poor sleep quality, short sleep duration, and long sleep duration by calculating the odds ratio (OR) with 95% confidence interval (CI). In the sensitivity analysis, we further divided the participants into three groups (<7, 7–9, >9 h) to assess factors associated with sleep duration <7 h and more than 9 h. Moreover, we additionally excluded participates with cognitive impairment in the multivariate logistic regression models to examine the robust of the results in the sensitivity analysis. All the analyses were performed with IBM SPSS 26.0 (IBM Corp., Armonk, NY, USA). Two-sided *p*-values < 0.05 indicated statistical significance.

## Results

### Basic characteristics of the research population

A total of 47,460 participants in the CLHLS survey from 2008 to 2018 were included in this study; the average age of the participants was 73.4 ± 6.5 years. Participants were divided into four groups by age: 32.61% were ≤79 years old, 27.17% were age 80–89 years, 24.69% were age 90–99 years, and 16.53% ≥100. A total of 44.09% of participants were men, and 55.91% were women. We found significant differences among groups for age group, marital status, residence (urban or rural), economic status, living arrangements, education level, number of chronic diseases, smoking, drinking, regular exercise, dietary diversity score subgroups, housework, outdoor activities, having pets or gardening, reading books and newspapers, raising poultry, playing cards, watching TV, and other factors (*p* < 0.001 for all; Supplementary Table 1).

### Trends and disparities in sleep quality among older people in China

**Figure 1:**
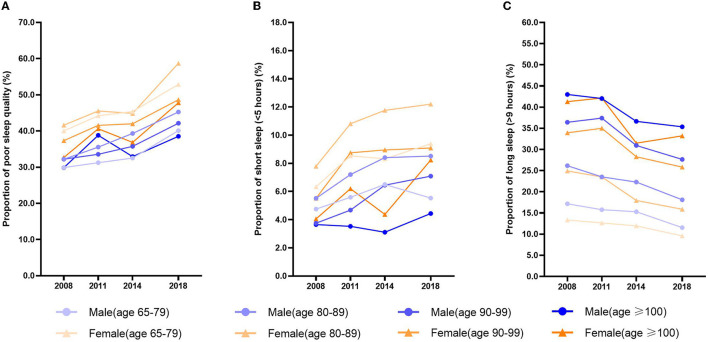
Trends of sleep quality and sleep duration in different gender and age groups.

The prevalence of poor sleep quality among older people in China increased over time, from 34.87% in 2008 to 47.67% in 2018 (*p* < 0.001, Table 1). Poor sleep quality showed the same upward trend across sex and age groups (Figure 1A). We found a higher prevalence of poor sleep quality among participants with characteristics such as female sex, age group 80–89 years, divorced or widowed, poor economic status, living in an institution, no education, ≥2 chronic diseases, no regular exercise, poor dietary diversity score, does not engage in reading, does not play cards, does not watch TV, no social participation, underweight, poor quality of life, poor health, and cognitive impairment (*p* < 0.001, Table 1).

**Table 1:** Trends and disparities in sleep quality among older people in China from 2008 to 2018.

	**Total**	**Good**	**Poor**	***p***
**Demographic factors**				
Investigation year				<0.001
2008	16,460	10,720 (65.13)	5,740 (34.87)	
2011	9,650	5,904 (61.18)	3,746 (38.82)	
2014	6,958	4,211 (60.52)	2,747 (39.48)	
2018	14,392	7,532 (52.33)	6,860 (47.67)	
Gender				<0.001
Male	20,924	13,465 (64.35)	7,459 (35.65)	
Female	26,536	14,902 (56.16)	11,634 (43.84)	
Age group				<0.001
≤79	15,005	9,022 (60.13)	5,983 (39.87)	
80–89	12,893	7,338 (56.91)	5,555 (43.09)	
90–99	11,719	7,127 (60.82)	4,592 (39.18)	
≥100	7,843	4,880 (62.22)	2,963 (37.78)	
Marital status				<0.001
Unmarried	438	247 (56.39)	191 (43.61)	
Married	17,691	10,826 (61.19)	6,865 (38.81)	
Divorced or widowed	29,030	17,114 (58.95)	11,916 (41.05)	
Category of residence				0.272
Urban (city and town)	22,328	13,287 (59.51)	9,041 (40.49)	
Rural	25,132	15,080 (60.00)	10,052 (40.00)	
**Socioeconomic status**				
Economic status				<0.001
Rich	7,717	5,473 (70.92)	2,244 (29.08)	
General	32,550	19,652 (60.37)	12,898 (39.63)	
Poor	6,748	3,022 (44.78)	3,726 (55.22)	
Living pattern				<0.001
Living with family members	38,098	23,156 (60.78)	14,942 (39.22)	
Living in an institution	1,183	604 (51.06)	579 (48.94)	
Living alone	7,812	4,392 (56.22)	3,420 (43.78)	
Years of schooling				<0.001
0	28,056	16,269 (57.99)	11,787 (42.01)	
≥1 year	19,404	12,098 (62.35)	7,306 (37.65)	
**Lifestyle habits**				
Smoking status				<0.001
Never	32,090	18,418 (57.39)	13,672 (42.61)	
Previous	7,138	4,498 (63.01)	2,640 (36.99)	
Current	7,851	5,225 (66.55)	2,626 (33.45)	
Alcohol intaking status				<0.001
Never	30,134	17,131 (56.85)	13,003 (43.15)	
Previous	4,688	2,799 (59.71)	1,889 (40.29)	
Current	7,851	5,225 (66.55)	2,626 (33.45)	
Regular exercise				<0.001
Never	28,039	16,149 (57.59)	11,890 (42.41)	
Previous	4,784	2,768 (57.86)	2,016 (42.14)	
Current	14,024	9,097 (64.87)	4,927 (35.13)	
Dietary diversity score				<0.001
Poor	14,774	7,531 (50.97)	7,243 (49.03)	
Moderate	24,039	15,041 (62.57)	8,998 (37.43)	
Good	8,561	5,747 (67.13)	2,814 (32.87)	
Housework				0.005
Almost everyday	19,937	11,769 (59.03)	8,168 (40.97)	
Sometimes	5,882	3,605 (61.29)	2,277 (38.71)	
Never	21,499	12,907 (60.04)	8,592 (39.96)	
Outdoor activities				<0.001
Almost everyday	18,812	12,115 (64.40)	6,697 (35.60)	
Sometimes	15,024	8,219 (54.71)	6,805 (45.29)	
Never	13,481	7,946 (58.94)	5,535 (41.06)	
Keeping pets or gardening				<0.001
Almost everyday	5,328	3,537 (66.39)	1,791 (33.61)	
Sometimes	2,606	1,635 (62.74)	971 (37.26)	
Never	39,389	23,112 (58.68)	16,277 (41.32)	
Reading books				<0.001
Almost everyday	4,954	3,343 (67.48)	1,611 (32.52)	
Sometimes	4,241	2,722 (64.18)	1,519 (35.82)	
Never	38,127	22,219 (58.28)	15,908 (41.72)	
Raising poultry				<0.001
Almost everyday	8,062	4,802 (59.56)	3,260 (40.44)	
Sometimes	2,797	1,633 (58.38)	1,164 (41.62)	
Never	36,455	21,841 (59.91)	14,614 (40.09)	
Playing cards				<0.001
Almost everyday	2,795	1,895 (67.80)	900 (32.20)	
Sometimes	4,658	2,909 (62.45)	1,749 (37.55)	
Never	39,869	23,475 (58.88)	16,394 (41.12)	
Watching TV				<0.001
Almost everyday	24,472	15,567 (63.61)	8,905 (36.39)	
Sometimes	8,371	4,695 (56.09)	3,676 (43.91)	
Never	14,487	8,029 (55.42)	6,458 (44.58)	
Social participation				<0.001
Almost everyday	1,294	876 (67.70)	418 (32.30)	
Sometimes	4,862	3,060 (62.94)	1,802 (37.06)	
Never	41,030	24,280 (59.18)	16,750 (40.82)	
**Health conditions**				
Numbers of chronic diseases				<0.001
0	17,291	11,610 (67.14)	5,681 (32.86)	
1	14,428	8,669 (60.08)	5,759 (39.92)	
≥2	14,917	7,712 (51.70)	7,205 (48.30)	
BMI				<0.001
Normal	26,798	16,375 (61.11)	10,423 (38.89)	
Underweight	10,937	6,168 (56.40)	4,769 (43.60)	
Overweight	5,943	3,695 (62.17)	2,248 (37.83)	
Obesity	1,265	758 (59.92)	507 (40.08)	
Activities of daily living				<0.001
Independent	35,455	21,615 (60.96)	13,840 (39.04)	
Disabled	10,872	6,111 (56.21)	4,761 (43.79)	
Self-reported quality of life				<0.001
Good	28,184	18,793 (66.68)	9,391 (33.32)	
General	13,553	6,655 (49.10)	6,898 (50.90)	
Poor	2,220	771 (34.73)	1,449 (65.27)	
Self-reported health				<0.001
Good	20,621	15,254 (73.97)	5,367 (26.03)	
General	16,473	8,307 (50.43)	8,166 (49.57)	
Poor	6,885	2,666 (38.72)	4,219 (61.28)	
Cognitive impairment				<0.001
No	35,743	21,686 (60.67)	14,057 (39.33)	
Yes	11,717	6,681 (57.02)	5,036 (42.98)	

### Trends and disparities in sleep duration among older people in China

Our study investigated changes in the proportion of sleep duration in the older people from 2008 to 2018. The proportion of short sleep duration (<5 h) increased, from 5.29% in 2008 to 8.37% in 2018. On the contrary, the proportion of long sleep duration (>9 h) decreased, from 28.77% in 2008 to 19.27% in 2018 (*p* < 0.001; Table 2). The same trend was observed across both sexes and across age groups (Figures 1B, C).

**Table 2:** Trends and disparities in sleep duration among older people in China from 2008 to 2018.

	**Total**	**<5 h**	**5-9 h**	**>9 h**	
Investigation year					<0.001
2008	16,405	868 (5.29)	10,818 (65.94)	4,719 (28.77)	
2011	9,574	703 (7.34)	6,340 (66.22)	2,531 (26.44)	
2014	6,897	547 (7.93)	4,857 (70.42)	1,493 (21.65)	
2018	14255	1,193 (8.37)	10,315 (72.36)	2,747 (19.27)	
Gender					<0.001
Male	20,804	1,210 (5.82)	14,689 (70.61)	4,905 (23.58)	
Female	26,327	2,101 (7.98)	17,641 (67.01)	6,585 (25.01)	
Age group					<0.001
≤79	14,926	1,009 (6.76)	11,942 (80.01)	1,975 (13.23)	
80–89	12,823	1,133 (8.84)	8,911 (69.49)	2,779 (21.67)	
90–99	11,621	764 (6.57)	7,112 (61.20)	3,745 (32.23)	
≥100	7,761	405 (5.22)	4,365 (56.24)	2,991 (38.54)	
Marital status					<0.001
Unmarried	431	40 (9.28)	288 (66.82)	103 (23.90)	
Married	17,605	1,194 (6.78)	13,361 (75.89)	3,050 (17.32)	
Divorced or widowed	28,796	2,054 (7.13)	18,475 (64.16)	8,267 (28.71)	
Category of residence of the interviewee at the 1998 survey					0.009
Urban (city and town)	22,193	1,514 (6.82)	15,376 (69.28)	5,303 (23.89)	
Rural	24,938	1,797 (7.21)	16,954 (67.98)	61,87 (24.81)	
Economic status					<0.001
Rich	7,674	349 (4.55)	5,390 (70.24)	1,935 (25.22)	
General	32,349	2,111 (6.53)	22,370 (69.15)	7,868 (24.32)	
Poor	6,671	814 (12.20)	4,294 (64.37)	1,563 (23.43)	
Living pattern					<0.001
Living with family members	37,866	2,445 (6.46)	25,914 (68.44)	9,507 (25.11)	
Living in a institution	1,169	117 (10.01)	748 (63.99)	304 (26.01)	
Living alone	7,738	721 (9.32)	5,410 (69.91)	1,607 (20.77)	
Years of schooling					<0.001
0	27,830	2,139 (7.69)	18,134 (65.16)	7,557 (27.15)	
≥1 year	19,301	1,172 (6.07)	14,196 (73.55)	3,933 (20.38)	
Numbers of chronic diseases					<0.001
0	17,169	839 (4.89)	11,676 (68.01)	4,654 (27.11)	
1	14,341	1,015 (7.08)	9,890 (68.96)	3,436 (23.96)	
≥2	14,827	1,389 (9.37)	10,211 (68.87)	3,227 (21.76)	
Smoking status					<0.001
Never	31,848	2,309 (7.25)	21,827 (68.53)	7,712 (24.22)	
Previous	7,090	477 (6.73)	4,777 (67.38)	1,836 (25.90)	
Current	7,817	501 (6.41)	5,475 (70.04)	1,841 (23.55)	
Alcohol intaking status					<0.001
Never	29,906	2,190 (7.32)	20,555 (68.73)	7,161 (23.95)	
Previous	4,662	364 (7.81)	3,010 (64.56)	1,288 (27.63)	
Current	7,817	501 (6.41)	5,475 (70.04)	1,841 (23.55)	
Regular exercise					<0.001
Never	27,829	1,971 (7.08)	18,713 (67.24)	7,145 (25.67)	
Previous	4,743	363 (7.65)	2,981 (62.85)	1,399 (29.50)	
Current	13,962	937 (6.71)	10,215 (73.16)	2,810 (20.13)	
Dietary diversity score					<0.001
Poor	14,622	1,445 (9.88)	9,702 (66.35)	3,475 (23.77)	
Moderate	23,900	1,525 (6.38)	16,294 (68.18)	6,081 (25.44)	
Good	8,525	337 (3.95)	6,276 (73.62)	1,912 (22.43)	
Housework					<0.001
Almost everyday	19,823	1,562 (7.88)	14,927 (75.30)	3,334 (16.82)	
Sometimes	5,854	322 (5.50)	4,358 (74.44)	1,174 (20.05)	
Never	21,314	1,421 (6.67)	12,957 (60.79)	6,936 (32.54)	
Outdoor activities					<0.001
Almost everyday	18,721	1,231 (6.58)	13,623 (72.77)	3,867 (20.66)	
Sometimes	14,910	1,095 (7.34)	10,544 (70.72)	3,271 (21.94)	
Never	13,360	978 (7.32)	8,077 (60.46)	4,305 (32.22)	
Keeping pets or gardening					<0.001
Almost everyday	5,297	320 (6.04)	4,092 (77.25)	885 (16.71)	
Sometimes	2,595	124 (4.78)	2,013 (77.57)	458 (17.65)	
Never	39,104	2,860 (7.31)	26,140 (66.85)	10,104 (25.84)	
Reading books					<0.001
Almost everyday	4,930	248 (5.03)	3,898 (79.07)	784 (15.90)	
Sometimes	4,219	226 (5.36)	3,247 (76.96)	746 (17.68)	
Never	37,846	2,830 (7.48)	25,099 (66.32)	9,917 (26.20)	
Raising poultry					<0.001
Almost everyday	8,018	567 (7.07)	6,041 (75.34)	1,410 (17.59)	
Sometimes	2,780	155 (5.58)	2,083 (74.93)	542 (19.50)	
Never	36,189	2,581 (7.13)	24,114 (66.63)	9,494 (26.23)	
Playing cards					<0.001
Almost everyday	2,785	162 (5.82)	2,120 (76.12)	503 (18.06)	
Sometimes	4,634	246 (5.31)	3,578 (77.21)	810 (17.48)	
Never	39,576	2,897 (7.32)	26,547 (67.08)	10,132 (25.60)	
Watching TV					<0.001
Almost everyday	24,360	1,567 (6.43)	18,084 (74.24)	4,709 (19.33)	
Sometimes	8,313	565 (6.80)	5,684 (68.37)	2,064 (24.83)	
Never	14,330	1,169 (8.16)	8,489 (59.24)	4,672 (32.60)	
Social participation					<0.001
Almost everyday	1,290	78 (6.05)	1,051 (81.47)	161 (12.48)	
Sometimes	4,829	274 (5.67)	3,866 (80.06)	689 (14.27)	
Never	40,741	2,942 (7.22)	27,231 (66.84)	10,568 (25.94)	
BMI					<0.001
Normal	26,658	1,789 (6.71)	18,741 (70.30)	6,128 (22.99)	
Underweight	10,860	816 (7.51)	7,005 (64.50)	3,039 (27.98)	
Overweight	5,916	387 (6.54)	4,377 (73.99)	1,152 (19.47)	
Obesity	1,257	96 (7.64)	891 (70.88)	270 (21.48)	
Activities of daily living					<0.001
Independent	35,254	2,429 (6.89)	25,536 (72.43)	7,289 (20.68)	
Disabled	10,758	790 (7.34)	6,027 (56.02)	3,941 (36.63)	
Self-reported quality of life					<0.001
Good	28,010	1,583 (5.65)	19,472 (69.52)	6,955 (24.83)	
General	13,478	1,183 (8.78)	9,691 (71.90)	2,604 (19.32)	
Poor	2,203	340 (15.43)	1,432 (65.00)	431 (19.56)	
Self-reported health					<0.001
Good	20,510	895 (4.36)	14,432 (70.37)	5,183 (25.27)	
General	16,373	1,259 (7.69)	11,833 (72.27)	3,281 (20.04)	
Poor	6,829	963 (14.10)	4,337 (63.51)	1,529 (22.39)	
Cognitive impairment					<0.001
No	35,562	2,487 (6.99)	25,969 (73.02)	7,106 (19.98)	
Yes	11,569	824 (7.12)	6,361 (54.98)	4,384 (37.89)	

Participants with the following characteristics had a higher prevalence of poor sleep duration: female sex, age 80–89 years, rural residence, poor economic status, living in an institution, ≥2 chronic diseases, poor dietary diversity score, does not engage in reading, does not play cards, never watches TV, no social participation, underweight, poor quality of life, poor health, and cognitive impairment (*p* < 0.001, Table 2).

### Multivariate logistic regression of factors associated with sleep quality

Multivariate logistic regression analysis showed that, after controlling for other confounding factors, the risk of poor sleep quality increased each year compared with 2008: adjusted odds ratio (aOR) = 1.08 (95% CI: 1.02–1.15) in 2011, aOR = 1.28 (95% CI: 1.19–1.38) in 2014, and aOR = 1.94 (95% CI: 1.82–2.05) in 2018 (Table 3). Female (aOR = 1.39, 95% CI: 1.30–1.48), poor economic status (aOR = 1.60, 95% CI: 1.46–1.76), ≥2 chronic diseases (aOR = 1.52, 95% CI: 1.43–1.61), underweight (aOR = 1.16, 95% CI: 1.08–1.24), no social participation (aOR = 1.32, 95% CI: 1.16–1.49), poor self-reported quality of life (aOR = 1.83, 95% CI: 1.63–2.06), and poor self-reported health (aOR = 2.56, 95% CI: 2.39–2.75) were related with poor sleep quality (*p* < 0.05). In the sensitivity analysis, the results were stable (Supplementary Table 2).

**Table 3:** Multivariate logistic regression analysis of poor sleep quality.

	**Beta**	**S.E**.	***p***	**OR**	**95% CI**
**Investigation year**					
2008				Ref.	
2011	0.079	0.032	0.014	1.08	1.02–1.15
2014	0.248	0.038	<0.001	1.28	1.19–1.38
2018	0.660	0.030	<0.001	1.94	1.82–2.05
**Gender**					
Male				Ref.	
Female	0.328	0.032	<0.001	1.39	1.30–1.48
**Marital status**					
Unmarried				Ref.	
Married	0.182	0.108	0.092	1.20	0.97–1.48
Divorced or widowed	0.266	0.108	0.013	1.30	1.06–1.61
**Category of residence**					
Rural				Ref.	
Urban (city and town)	−0.060	0.025	0.016	0.94	0.90–0.99
**Economic status**					
Rich				Ref.	
General	0.177	0.034	<0.001	1.19	1.12–1.28
Poor	0.471	0.048	<0.001	1.60	1.46–1.76
**Numbers of chronic diseases**					
0				Ref.	
1	0.131	0.029	<0.001	1.14	1.08–1.21
≥2	0.417	0.029	<0.001	1.52	1.43–1.61
**Smoking status**					
Never				Ref.	
Previous	−0.019	0.040	0.630	0.98	0.91–1.06
Current	−0.173	0.034	<0.001	0.84	0.79–0.90
**Regular exercise**					
Never				Ref.	
Previous	0.191	0.045	<0.001	1.21	1.11–1.32
Current	0.010	0.027	0.725	1.01	0.96–1.06
**Dietary diversity score**					
Poor				Ref.	
Moderate	−0.246	0.027	<0.001	0.78	0.74–0.83
Good	−0.192	0.036	<0.001	0.83	0.77–0.88
**Housework**					
Almost everyday				Ref.	
Sometimes	0.110	0.035	0.002	1.12	1.04–1.20
Never	−0.031	0.034	0.363	0.97	0.91–1.04
**Outdoor activities**					
Almost everyday				Ref.	
Sometimes	0.185	0.027	<0.001	1.20	1.14–1.27
Never	0.004	0.035	0.913	1.00	0.94–1.08
**Keeping pets or gardening**					
Almost everyday				Ref.	
Sometimes	0.022	0.049	0.662	1.02	0.93–1.13
Never	0.115	0.032	<0.001	1.12	1.05–1.20
**Reading books**					
Almost everyday				Ref.	
Sometimes	−0.208	0.044	<0.001	0.81	0.74–0.89
Never	−0.075	0.039	0.054	0.93	0.86–1.00
**Raising poultry**					
Almost everyday				Ref.	
Sometimes	−0.029	0.048	0.550	0.97	0.88–1.07
Never	−0.095	0.028	0.001	0.91	0.86–0.96
**Watching TV**					
Almost everyday				Ref.	
Sometimes	0.131	0.032	<0.001	1.14	1.07–1.21
Never	0.196	0.037	<0.001	1.22	1.13–1.31
**Social participation**					
Almost everyday				Ref.	
Sometimes	0.354	0.067	<0.001	1.43	1.25–1.62
Never	0.274	0.064	<0.001	1.32	1.16–1.49
**BMI**					
Normal				Ref.	
Underweight	0.146	0.033	<0.001	1.16	1.08–1.24
Overweight	−0.206	0.030	<0.001	0.81	0.77–0.86
Obesity	−0.242	0.061	<0.001	0.79	0.70–0.89
**Activities of daily living**					
Independent				Ref.	
Disabled	−0.110	0.050	0.027	0.90	0.81–0.99
**Self–reported quality of life**					
Good				Ref.	
General	0.446	0.026	<0.001	1.56	1.48–1.64
Poor	0.604	0.060	<0.001	1.83	1.63–2.06
**Self–reported health**					
Good				Ref.	
General	0.800	0.026	<0.001	2.23	2.11–2.34
Poor	0.942	0.037	<0.001	2.56	2.39–2.75

### Multivariate logistic regression of factors associated with sleep duration

Multivariate logistic regression analysis showed that, after controlling for other confounding factors, the risk of short sleep duration increased each year compared with 2008 (Table 4): aOR = 1.25 (95% CI: 1.11–1.42) in 2011, aOR = 1.52 (95% CI: 1.32–1.76) in 2014, and aOR = 1.69 (95% CI: 1.50–1.89) in 2018.

**Table 4:** Multivariate logistic regression of short sleep duration and long sleep duration.

	**Risk factors of short sleep duration**			**Risk factors of long sleep duration**		
	**OR**	***p***	**95% CI**	**OR**	***p***	**95% CI**
**Investigation year**						
2008	Ref.			Ref.		
2011	1.25	<0.001	1.11–1.42	0.98	0.635	0.91–1.06
2014	1.52	<0.001	1.32–1.76	0.92	0.096	0.84–1.01
2018	1.69	<0.001	1.50–1.89	0.65	<0.001	0.60–0.71
**Gender**						
Male	Ref.			Ref.		
Female	1.44	<0.001	1.28–1.62	0.77	<0.001	0.71–0.84
**Age group**						
≤79	Ref.			Ref.		
80–89	1.36	<0.001	1.22–1.52	1.42	<0.001	1.31–1.54
90–99	1.18	0.329	0.85–1.64	1.90	<0.001	1.57–2.31
≥100	0.75	0.838	0.05–11.73	1.94	0.276	0.59–6.43
**BMI**						
Normal	Ref.			Ref.		
Underweight	1.18	0.005	1.05–1.33	0.96	0.343	0.88–1.05
Overweight	0.99	0.906	0.89–1.11	1.10	0.015	1.02–1.19
Obesity	0.85	0.180	0.67–1.08	1.14	0.114	0.97–1.34
**Marital status**						
Unmarried				Ref.		
Married				0.41	<0.001	0.33–0.51
Divorced or widowed				0.44	<0.001	0.35–0.55
**Economic status**						
Rich	Ref.	<0.001				
General	1.18	0.032	1.01–1.37			
Poor	1.91	<0.001	1.60–2.28			
**Living pattern**						
Living with family members	Ref.			Ref.		
Living in a institution	1.74	0.001	1.24–2.45	0.92	0.545	0.70–1.21
Living alone	1.06	0.303	0.95–1.18	0.88	0.009	0.80–0.97
**Years of schooling**						
0	Ref.			Ref.		
≥1 year	0.77	<0.001	0.69–0.84	0.83	<0.001	0.77–0.88
**Numbers of chronic diseases**						
0	Ref.					
1	1.34	<0.001	1.19–1.51			
≥2	1.68	<0.001	1.50–1.88			
**Smoking status**						
Never	Ref.			Ref.		
Previous	1.38	<0.001	1.19–1.60	1.23	<0.001	1.12–1.36
Current	1.30	<0.001	1.14–1.48	1.10	0.035	1.01–1.19
**Regular exercise**						
Never	Ref.			Ref.		
Previous	1.18	0.047	1.00–1.40	1.20	0.001	1.07–1.33
Current	1.25	<0.001	1.13–1.38	0.98	0.567	0.91–1.05
**Dietary diversity score**						
Poor	Ref.			Ref.		
Moderate	0.82	<0.001	0.74–0.90	1.07	0.067	1.00–1.15
Good	0.50	<0.001	0.43–0.59	0.91	0.039	0.83–1.00
**Housework**						
Almost everyday				Ref.		
Sometimes				1.06	0.216	0.97–1.16
Never				1.29	<0.001	1.18–1.39
**Outdoor activities**						
Almost everyday	Ref.			Ref.		
Sometimes	0.89	0.022	0.80–0.98	0.92	0.025	0.85–0.99
Never	0.86	0.023	0.75–0.98	0.97	0.528	0.89–1.06
**Keeping pets or gardening**						
Almost everyday				Ref.		
Sometimes				0.71	<0.001	0.62–0.81
Never				0.86	0.001	0.79–0.94
**Reading books**						
Almost everyday	Ref.			Ref.		
Sometimes	0.96	0.644	0.79–1.16	1.42	<0.001	1.25–1.60
Never	1.15	0.088	0.98–1.35	1.46	<0.001	1.31–1.62
**Raising poultry**						
Almost everyday				Ref.	0.003	
Sometimes				0.81	0.002	0.71–0.93
Never				1.00	0.913	0.94-1.08
**Watching TV**						
Almost everyday	Ref.			Ref.		
Sometimes	0.92	0.156	0.81–1.03	1.19	<0.001	1.10–1.29
Never	1.24	<0.001	1.10–1.40	1.40	<0.001	1.28–1.53
**Social participation**						
Almost everyday				Ref.		
Sometimes				1.59	<0.001	1.29–1.96
Never				2.05	<0.001	1.68–2.50
**Activities of daily living**						
Independent				Ref.		
Disabled				1.45	<0.001	1.29–1.62
**Self–reported quality of life**						
Good	Ref.			Ref.		
General	1.39	<0.001	1.26–1.53	0.83	<0.001	0.78–0.89
Poor	1.64	<0.001	1.38–1.95	0.78	0.002	0.67–0.92
**Self–reported health**						
Good	Ref.			Ref.		
General	1.54	<0.001	1.38–1.72	0.86	<0.001	0.80–0.92
Poor	2.30	<0.001	2.02–2.62	0.99	0.819	0.90–1.09

Female sex (aOR = 1.44, 95% CI: 1.28–1.62), poor economic status (aOR = 1.91, 95% CI: 1.60–2.28), ≥2 chronic diseases (aOR = 1.68, 95% CI: 1.50–1.88), underweight (aOR = 1.18, 95% CI: 1.05–1.33), current smoking (aOR = 1.30, 95% CI: 1.14–1.48), living in an institution (aOR = 1.74, 95% CI: 1.24–2.45), poor self-reported quality of life (aOR = 1.64,95% CI: 1.38–1.95), and poor self-reported health (aOR = 2.30, 95% CI: 2.02–2.62) were risk factors of short sleep duration (p < 0.05). Good dietary diversity score (aOR = 0.50, 95% CI: 0.43–0.59) and ≥1 year of schooling (aOR = 0.77, 95% CI: 0.69–0.84) were protective factors against short sleep duration (*p* < 0.05, Table 4).

In contrast to short sleep duration, the odds of long sleep duration were not increased in 2011 and 2014; however, the likelihood of long sleep duration was decreased in 2018 compared with 2008: aOR = 0.98 (95% CI: 0.91–1.06) in 2011, aOR = 0.92 (95% CI: 0.84–1.01) in 2014, and aOR = 0.65 (95% CI: 0.60–0.71) in 2018.

In the sensitivity analysis, similar results were found in the models (Supplementary Tables 3, 4).

## Discussion

Our study showed that the prevalence of self-reported poor sleep quality among older people increased from one in three to nearly one in two between 2008 and 2018 in China. Meanwhile, the prevalence of short sleep duration increased from 5.29 to 8.37%, whereas the prevalence of long sleep duration decreased from 28.77 to 19.27%. Previous studies have investigated the prevalence of poor sleep quality among older people in China, showing rates ranging from 33.8 to 49.7% (8–11), which is similar to our study. We also compared sleep quality in our population with that reported in other countries, which is 28.9% in Japan (2) and 17.8% in Brazil (35). It can be seen that the prevalence of poor sleep quality is generally high in older age groups, although there are differences worldwide. The differences between our study findings and other research results may be owing to the differences of age groups, sample sizes, interviewing techniques, economic levels at regional or country level, and culture. Owing to the lack of reports on trends of sleep quality and duration among older people, we compared our findings with trends of sleep duration among adults in the United States during a similar period (3). Consistent with our results, the prevalence of insufficient sleep was shown to be increasing, accompanied by a decline in the proportion of long sleep duration in the United States (3). The trend changes observed in our study population may be related to rapid social and economic development in China during the study period, with an accelerating pace of life and increasing life pressure (36). Our findings supplemented more evidence on the trend of poor sleep quality and short sleep duration, more importantly, indicated sleep health problems among older people was becoming serious. Therefore, providing the possible influencing factors to improve sleep is crucial.

According to our study, female sex was an independent risk factor for poor sleep quality and insufficient sleep duration in older people, which is consistent with study in China (10), Sweden (37) and Korea (38). Women are more likely to have depression (22) and report more severe physical conditions than men (39), so their sleep may be affected easier. Additionally, in traditional Chinese culture, women have a lower status in the family and do more housework, which may also be influencing factors for poorer sleep. In fact, the role of caregiver for women in most cases, means s the multiple tasks, generates stress and affect sleep. In this study, lower BMI was associated with poor sleep quality, but obesity and overweight were not significantly associated with poor sleep quality or abnormal sleep duration. Although some studies found that obesity was associated with poor sleep quality (40–42) which was not consistent with our findings, one study found that the higher BMI was, the more better sleep quality was in Chinese men which partly supported our findings (43). We attribute these contradictory results to China's unique conditions, in which a higher BMI is associated with better socioeconomic level, better living conditions, and less economic stress; these in turn may lead to better sleep quality. In addition, Tang et al. (22) found that the greater BMI was a protective factor for depression, while disturbed sleep was a risk factor and the depression. Gu et al. (44) found that participants with BMI <18.5 kg/m^2^ were at a significantly higher risk of frailty than those within the normal BMI range, while frailty was associated with sleep problems (45). Depression and frailty may be the possible mediating factors for the relationship between BMI and sleep, butthe truth of this remains should be investigated in the future.

We found that never participating in social activities were associated with long sleep duration and poor sleep quality. Consistent with previous research, long sleep duration showed an inverse association with engaging in social activities (46, 47). In a cross-sectional study among older people, participation in social activities was associated with better sleep quality (48). Generally, for older people who are retired, no longer accompanied by relatives and less physically active, participating in social activities adds to having more social capital, which helps to promote the physical health of older people (49) and prevent depression (50). We found that not engaging in reading was associated with long sleep duration and not watching TV were associated with long sleep duration and poor sleep quality. Dzierzewski et al. (51) and Xie et al. (52) both found that excessive the duration of watching TV predicted poorer sleep status among older people. Our findings further provided that never watching TV was also harmful to sleep, not only excessive the duration of watching TV. Combining our findings and previous studies, moderate leisure activities would be acceptable. Similarly, leisure activities (reading, watching TV) also benefit for preventing depression among older people (53). Therefore, developing healthy living habits that include active participation in social activities and developing new interests may be one way to improve sleep quality and sleep duration among older people in China.

Large sample data and the inclusion of demographic factors, socioeconomic status, lifestyle habits, and health condition variables are among the strengths of this study. However, there are some study limitations as well. Limited information about self-reported sleep duration and sleep quality was the primary drawback in this study, although the reliability and validity of self-reported sleep duration questionnaires have been demonstrated (54). Besides, non-objective reporting may not distinguish between time spent in bed and time actually asleep. Future research should consider the use of objective methods in sleep assessment, such as using smart devices to collect sleep information (55). The questionnaire only included one question about sleeping time and did not make a detailed distinction of the period, such as weekend or weekday, which is considered to be different in the literature (56). The questionnaire also did not directly investigate the psychological status of older people, which may also be a risk factor in their sleep quality and sleep duration (11). Finally, the subjects of each wave are the older people who meet the criteria, so participants were different across study waves. Therefore, the data may be biased, and more scientific evidence is needed in the future.

## Conclusion

In the past 10 years, the number of older people in China with poor sleep quality has increased from one-third to nearly one-half. The proportion of older adults with short sleep duration increased from 2008 to 2018 whereas the proportion with long sleep duration decreased. Female sex, poor economic status, low social participation, low BMI, and the number of chronic diseases were risk factors associated with sleep quality and duration. China is an aging society, and public health officials must pay attention to the sleep status of older people. At present, the literature on trends and disparities of sleep in the older people in China is limited. Our study has important value in guiding sleep health care among the older people in China.

## Data availability statement

Publicly available datasets were analyzed in this study. This data can be found here: https://opendata.pku.edu.cn/dataverse/CHADS;jsessionid=c49bc1a85ef56a3e899accc581b8.

## Ethics statement

The studies involving human participants were reviewed and approved by the Ethical Review Committee of Peking University (IRB00001052-13074). The patients/participants provided their written informed consent to participate in this study.

## Author contributions

Conception and design: LT and JL. Administrative support: JL. Provision of study materials or patients: ZT and YF. Collection and assembly of data: YF. Data analysis and interpretation: ZT and LT. Manuscript writing and final approval of manuscript: All authors.
