# Supplementary material for: Trends and disparities in sleep quality and duration in older adults in China from 2008 to 2018: A national observational study
Source: Front Public Health. 2023 Feb 17;11:998699. doi: 10.3389/fpubh.2023.998699 (PMC9982158; doi:10.3389/fpubh.2023.998699)
Supplement: Supplementary file 1 [file Data_Sheet_1.pdf]

Supplementary Table 1. Basic characteristics of the research population

	2008	2011	2014	2018	total	p
Gender						<0.001
Male	6957(42.27)	4322(44.79)	3188(45.82)	6457(44.87)	20924(44.09)	
Female	9503(57.73)	5328(55.21)	3770(54.18)	7935(55.13)	26536(55.91)	
Age group						<0.001
<=79	4280(26.00)	3143(32.57)	2324(33.40)	5258(36.53)	15005(31.62)	
80-89	4268(25.93)	2634(27.30)	2166(31.13)	3825(26.58)	12893(27.17)	
90-99	4571(27.77)	2425(25.13)	1612(23.17)	3111(21.62)	11719(24.69)	
>=100	3341(20.30)	1448(15.01)	856(12.30)	2198(15.27)	7843(16.53)	
Marital status						<0.001
Unmarried	147(0.89)	99(1.03)	69(1.00)	123(0.86)	438(0.93)	
Married	5175(31.44)	3616(37.77)	2728(39.66)	6172(43.32)	17691(37.51)	
Divorced or widowed	11138(67.67)	5858(61.19)	4081(59.33)	7953(55.82)	29030(61.56)	
Category of residence						<0.001
Urban (city and town)	6582(39.99)	4586(47.52)	3157(45.37)	8003(55.61)	22328(47.05)	
Rural	9878(60.01)	5064(52.48)	3801(54.63)	6389(44.39)	25132(52.95)	
Economic status						<0.001
Rich	2169(13.21)	1629(17.14)	1108(16.18)	2811(19.73)	7717(16.41)	
General	11256(68.58)	6391(67.25)	4946(72.21)	9957(69.88)	32550(69.23)	
Poor	2989(18.21)	1484(15.61)	795(11.61)	1480(10.39)	6748(14.35)	
Living pattern						<0.001
Living with family members	13619(82.74)	7714(80.83)	5404(78.22)	11361(80.12)	38098(80.90)	
Living in an institution	304(1.85)	206(2.16)	198(2.87)	475(3.35)	1183(2.51)	
Living alone	2537(15.41)	1624(17.02)	1307(18.92)	2344(16.53)	7812(16.59)	
Years of schooling						<0.001
0	10430(63.37)	5655(58.60)	3986(57.29)	7985(55.48)	28056(59.12)	
>=1 year	6030(36.63)	3995(41.40)	2972(42.71)	6407(44.52)	19404(40.88)	
Numbers of chronic diseases						<0.001
0	7059(42.89)	3596(37.82)	2398(35.55)	4238(30.44)	17291(37.08)	
1	5107(31.03)	2802(29.47)	2124(31.49)	4395(31.57)	14428(30.94)	
>=2	4294(26.09)	3111(32.72)	2223(32.96)	5289(37.99)	14917(31.99)	
Smoking status						<0.001
Never	11074(67.31)	6310(66.02)	4876(70.43)	9830(69.48)	32090(68.16)	
Previous	2577(15.66)	1537(16.08)	909(13.13)	2115(14.95)	7138(15.16)	
Current	2801(17.03)	1710(17.89)	1138(16.44)	2202(15.57)	7851(16.68)	
Alcohol intaking status						<0.001
Never	10259(68.94)	5794(67.49)	4655(73.78)	9426(73.08)	30134(70.62)	
Previous	1820(12.23)	1081(12.59)	516(8.18)	1271(9.85)	4688(10.99)	
Current	2801(18.82)	1710(19.92)	1138(18.04)	2202(17.07)	7851(18.40)	
Regular exercise						<0.001
Never	9731(59.17)	5154(54.33)	4593(67.76)	8561(60.56)	28039(59.85)	
Previous	2186(13.29)	1169(12.32)	369(5.44)	1060(7.50)	4784(10.21)	

Current	4530(27.54)	3163(33.34)	1816(26.79)	4515(31.94)	14024(29.94)	
Dietary diversity score						<0.001
Poor	4808(29.21)	3292(34.28)	2191(31.54)	4483(31.21)	14774(31.19)	
Moderate	8498(51.63)	4804(50.03)	3625(52.18)	7112(49.51)	24039(50.74)	
Good	3154(19.16)	1506(15.68)	1131(16.28)	2770(19.28)	8561(18.07)	
Housework						<0.001
Almost everyday	6248(37.96)	4186(43.62)	3197(46.13)	6306(44.01)	19937(42.13)	
Sometimes	2334(14.18)	1091(11.37)	780(11.25)	1677(11.70)	5882(12.43)	
Never	7878(47.86)	4320(45.01)	2954(42.62)	6347(44.29)	21499(45.44)	
Outdoor activities						<0.001
Almost everyday	6504(39.51)	4071(42.42)	2871(41.40)	5366(37.46)	18812(39.76)	
Sometimes	3470(21.08)	1692(17.63)	1226(17.68)	8636(60.29)	15024(31.75)	
Never	6486(39.40)	3835(39.96)	2837(40.91)	323(2.25)	13481(28.49)	
Keeping pets or gardening						<0.001
Almost everyday	1245(7.56)	1309(13.64)	959(13.85)	1815(12.66)	5328(11.26)	
Sometimes	799(4.85)	574(5.98)	430(6.21)	803(5.60)	2606(5.51)	
Never	14416(87.58)	7712(80.38)	5537(79.95)	11724(81.75)	39389(83.23)	
Reading books						<0.001
Almost everyday	1433(8.71)	1076(11.22)	733(10.58)	1712(11.94)	4954(10.47)	
Sometimes	1250(7.59)	843(8.79)	661(9.54)	1487(10.37)	4241(8.96)	
Never	13777(83.70)	7672(79.99)	5534(79.88)	11144(77.70)	38127(80.57)	
Raising poultry						<0.001
Almost everyday	2547(15.47)	1713(17.85)	1355(19.57)	2447(17.07)	8062(17.04)	
Sometimes	1183(7.19)	551(5.74)	376(5.43)	687(4.79)	2797(5.91)	
Never	12730(77.34)	7330(76.40)	5192(75.00)	11203(78.14)	36455(77.05)	
Playing cards						<0.001
Almost everyday	825(5.01)	582(6.07)	450(6.50)	938(6.54)	2795(5.91)	
Sometimes	1493(9.07)	902(9.40)	727(10.50)	1536(10.71)	4658(9.84)	
Never	14142(85.92)	8108(84.53)	5748(83.00)	11871(82.75)	39869(84.25)	
Watching TV						<0.001
Almost everyday	7538(45.80)	5018(52.30)	3763(54.29)	8153(56.84)	24472(51.71)	
Sometimes	3335(20.26)	1570(16.36)	1162(16.77)	2304(16.06)	8371(17.69)	
Never	5587(33.94)	3007(31.34)	2006(28.94)	3887(27.10)	14487(30.61)	
Social participation						<0.001
Almost everyday	403(2.45)	277(2.89)	211(3.05)	403(2.83)	1294(2.74)	
Sometimes	1470(8.93)	1055(11.01)	753(10.88)	1584(11.14)	4862(10.30)	
Never	14587(88.62)	8253(86.10)	5956(86.07)	12234(86.03)	41030(86.95)	
BMI						<0.001
Normal	9370(58.28)	5297(59.47)	3983(62.84)	8148(59.82)	26798(59.63)	
Underweight	5329(33.15)	2231(25.05)	1178(18.59)	2199(16.14)	10937(24.34)	
Overweight	1191(7.41)	1069(12.00)	955(15.07)	2728(20.03)	5943(13.22)	
Obesity	187(1.16)	310(3.48)	222(3.50)	546(4.01)	1265(2.81)	
Activities of daily living						<0.001
Independent	12805(77.80)	6880(73.37)	5046(75.37)	10724(77.73)	35455(76.53)	
Disabled	3654(22.20)	2497(26.63)	1649(24.63)	3072(22.27)	10872(23.47)	
Self-reported quality of life						<0.001
Good	8601(59.29)	5410(60.81)	4314(66.65)	9859(70.01)	28184(64.12)	
General	4851(33.44)	3009(33.82)	1916(29.60)	3777(26.82)	13553(30.83)	

Poor	1054(7.27)	477(5.36)	243(3.75)	446(3.17)	2220(5.05)	
Self-reported health						<0.001
Good	7117(49.06)	4007(44.98)	2857(44.17)	6640(47.10)	20621(46.89)	
General	5018(34.59)	3345(37.55)	2609(40.34)	5501(39.02)	16473(37.46)	
Poor	2371(16.34)	1556(17.47)	1002(15.49)	1956(13.88)	6885(15.66)	

Supplemental Table 2. Multivariate logistic regression analysis of poor sleep quality

	Beta	S.E.	p	OR	95% CI
Investigation year					
2008				ref.	
2011	0.130	0.035	<0.001	1.14	1.06-1.22
2014	0.218	0.040	<0.001	1.24	1.15-1.34
2018	0.654	0.034	<0.001	1.92	1.80-2.06
Gender					
Male				ref.	
Female	0.284	0.031	<0.001	1.33	1.25-1.41
Marital status					
Unmarried				ref.	
Married	0.150	0.127	0.240	1.16	0.90-1.49
Divorced or widowed	0.159	0.128	0.013	1.17	1.01-1.31
Category of residence					
Rural				ref.	
Urban (city and town)	-0.059	0.027	0.026	0.94	0.89-0.99
Economic status					
Rich				ref.	
General	0.231	0.035	<0.001	1.26	1.18-1.35
Poor	0.529	0.050	<0.001	1.70	1.54-1.87
Numbers of chronic diseases					
0				ref.	
1	0.184	0.031	<0.001	1.20	1.13-1.28
>=2	0.441	0.031	<0.001	1.55	1.46-1.65
Smoking status					
Never				ref.	
Previous	-0.108	0.038	0.004	0.90	0.83-0.97
Current	-0.165	0.036	<0.001	0.85	0.79-0.91
Regular exercise					
Never				ref.	
Previous	0.071	0.045	0.118	1.07	0.98-1.17
Current	0.008	0.030	0.779	1.01	0.95-1.07
Dietary diversity score					
poor				ref.	
Moderate	-0.241	0.029	<0.001	0.79	0.74-0.83
Good	-0.252	0.039	<0.001	0.78	0.72-0.84
Housework					
Almost everyday				ref.	
Sometimes	-0.024	0.038	0.535	0.98	0.91-1.05
Never	-0.073	0.031	0.021	0.93	0.87-0.99
Outdoor activities					
Almost everyday				ref.	
Sometimes	0.114	0.031	<0.001	1.12	1.06-1.19

Never	0.098	0.037	0.008	1.10	1.03-1.19
Keeping pets or gardening					
Almost everyday				ref.	
Sometimes	0.093	0.059	0.118	1.10	0.98-1.23
Never	0.131	0.039	0.001	1.14	1.06-1.23
Reading books					
Almost everyday				ref.	
Sometimes	-0.049	0.052	0.349	0.95	0.86-1.05
Never	0.022	0.043	0.607	1.02	0.94-1.11
Raising poultry					
Almost everyday				ref.	
Sometimes	0.008	0.056	0.880	1.01	0.90-1.13
Never	-0.098	0.033	0.003	0.91	0.85-0.97
Watching TV					
Almost everyday				ref.	
Sometimes	0.151	0.034	<0.001	1.16	1.09-1.24
Never	0.071	0.035	0.042	1.07	1.00-1.15
Social participation					
Almost everyday				ref.	
Sometimes	0.097	0.078	0.211	1.10	0.95-1.28
Never	0.045	0.073	0.533	1.05	0.91-1.21
BMI					
Normal				ref.	
Underweight	0.146	0.032	<0.001	1.16	1.09-1.23
Overweight	-0.125	0.036	0.001	0.88	0.82-0.95
Obesity	-0.027	0.073	0.706	0.97	0.84-1.12
Activities of daily living					
Independent				ref.	
Disabled	-0.072	0.041	0.080	0.93	0.86-1.01
Self-reported quality of life					
Good				ref.	
General	0.354	0.029	<0.001	1.42	1.35-1.51
Poor	0.542	0.067	<0.001	1.72	1.51-1.96
Self-reported health					
Good				ref.	
General	0.812	0.028	<0.001	2.25	2.13-2.38
Poor	1.050	0.041	<0.001	2.86	2.64-3.10

Notes: Excluded participants with cognitive impairment.

Supplemental Table 3. Multivariate logistic regression of sleep duration.

	Risk factors of short sleep duration (<5h)			Risk factors of long sleep duration (>9h)		
	OR	p	95% CI	OR	p	95% CI
Investigation year						
2008	ref.			ref.		
2011	1.29	<0.001	1.13-1.47	0.95	0.170	0.88-1.02
2014	1.47	<0.001	1.27-1.69	0.83	<0.001	0.76-0.91
2018	1.58	<0.001	1.39-1.79	0.69	<0.001	0.64-0.75
Gender						
Male	ref.			ref.		
Female	1.38	<0.001	1.23-1.55	0.82	<0.001	0.76-0.88
Age group						
<=79	ref.			ref.		
80-89	1.32	<0.001	1.19-1.47	1.54	<0.001	1.42-1.66
90-99	1.17	0.024	1.02-1.33	2.15	<0.001	1.97-2.35
>=100	0.93	0.499	0.75-1.15	2.41	<0.001	2.14-2.70
BMI						
Normal	ref.			ref.		
Underweight	1.14	0.019	1.02-1.28	1.00	0.960	0.93-1.08
Overweight	0.88	0.063	0.77-1.01	1.05	0.321	0.96-1.14
Obesity	1.07	0.568	0.84-1.38	1.05	0.577	0.88-1.26
Marital status						
Unmarried				Ref.		
Married				0.75	0.045	0.56-0.98
Divorced or widowed				0.84	0.251	0.63-1.13
Economic status						
Rich	Ref.					
General	1.19	0.018	1.03-1.36			
Poor	1.71	<0.001	1.44-2.05			
Living pattern						
Living with family members	Ref.			Ref.		
Living in an institution	1.67	<0.001	1.26-2.22	0.99	0.949	0.81-1.22
Living alone	1.17	0.006	1.05-1.30	0.89	0.004	0.82-0.96
Years of schooling						
0				Ref.		
>=1 year				0.95	0.170	0.89-1.02
Numbers of chronic diseases						
0	Ref.					
1	1.25	<0.001	1.11-1.41			
>=2	1.53	<0.001	1.36-1.72			
Smoking status						

Never	Ref.			Ref.		
Previous	1.19	0.012	1.04-1.37	1.19	<0.001	1.10-1.30
Current	1.19	0.011	1.04-1.36	1.27	<0.001	1.17-1.38
Regular exercise						
Never	Ref.			Ref.		
Previous	1.19	0.029	1.02-1.40	1.17	0.032	1.01-1.32
Current	1.24	<0.001	1.11-1.38	0.96	0.277	0.90-1.03
Dietary diversity score						
Poor	Ref.			Ref.		
Moderate	0.81	<0.001	0.73-0.89	1.06	0.082	0.99-1.14
Good	0.52	<0.001	0.45-0.61	0.97	0.567	0.89-1.07
Outdoor activities						
Almost everyday	Ref.			Ref.		
Sometimes	0.94	0.279	0.84-1.05	0.90	0.006	0.84-0.97
Never	1.10	0.145	0.97-1.26	0.98	0.558	0.90-1.06
Keeping pets or gardening						
Almost everyday	Ref.			Ref.		
Sometimes	0.77	0.033	0.61-0.98	0.96	0.572	0.83-1.11
Never	1.02	0.733	0.89-1.18	0.98	0.716	0.90-1.08
Reading books						
Almost everyday	Ref.			Ref.		
Sometimes	1.03	0.778	0.84-1.26	1.23	0.001	1.09-1.40
Never	1.16	0.081	0.98-1.37	1.32	<0.001	1.19-1.46
Raising poultry						
Almost everyday				Ref.	0.003	
Sometimes				0.91	0.183	0.80-1.04
Never				1.04	0.330	0.96-1.12
Watching TV						
Almost everyday				Ref.		
Sometimes				1.17	<0.001	1.09-1.27
Never				1.28	<0.001	1.19-1.39
Social participation						
Almost everyday				Ref.		
Sometimes				1.17	0.144	0.95-1.44
Never				1.64	<0.001	1.35-1.99
Activities of daily living						
Independent				Ref.		
Disabled				1.41	<0.001	1.29-1.53
Self-reported quality of life						
Good	Ref.			Ref.		
General	1.15	0.010	1.03-1.27	0.77	<0.001	0.71-0.82
Poor	1.35	0.002	1.11-1.64	0.68	<0.001	0.58-0.80
Self-reported health						
Good	Ref.			Ref.		

General	1.47	<0.001	1.32-1.64	0.80	<0.001	0.74-0.85
Poor	2.29	<0.001	2.00-2.62	1.01	0.832	0.92-1.11

Notes: Excluded participants with cognitive impairment.

Supplemental Table 4. Multivariate logistic regression of sleep duration.

	Risk factors of short sleep duration (<7h)			Risk factors of long sleep duration (>9h)		
	OR	p	95% CI	OR	p	95% CI
Investigation year						
2008	ref.			ref.		
2011	1.24	<0.001	1.16-1.33	1.01	0.724	0.94-1.09
2014	1.25	<0.001	1.16-1.35	0.82	<0.001	0.75-0.90
2018	1.57	<0.001	1.47-1.67	0.79	<0.001	0.73-0.85
Gender						
Male	ref.			ref.		
Female	1.26	<0.001	1.19-1.34	0.92	0.026	0.86-0.99
Age group						
<=79	ref.			ref.		
80-89	1.19	<0.001	1.12-1.26	1.61	<0.001	1.49-1.74
90-99	1.17	<0.001	1.09-1.26	2.17	<0.001	1.99-2.37
>=100	1.08	0.086	0.99-1.19	2.27	<0.001	2.04-2.53
BMI						
Normal	ref.			ref.		
Underweight	1.02	0.617	0.96-1.08	1.00	0.954	0.94-1.07
Overweight	0.96	0.225	0.89-1.03	1.05	0.236	0.97-1.15
Obesity	0.99	0.869	0.86-1.14	1.06	0.528	0.89-1.25
Marital status						
Unmarried				Ref.		
Married				0.63	0.002	0.47-0.84
Divorced or widowed				0.72	0.023	0.54-0.96
Economic status						
Rich	Ref.					
General	1.11	0.002	1.04-1.19			
Poor	1.38	<0.001	1.25-1.52			
Living pattern						
Living with family members	Ref.			Ref.		
Living in a institution	1.30	0.002	1.10-1.53	1.05	0.580	0.88-1.27
Living alone	1.09	0.009	1.02-1.16	0.95	0.186	0.87-1.03
Years of schooling						
0	Ref.			Ref.		
>=1 year	0.95	0.040	0.92-0.98	0.96	0.022	0.90-0.99
Numbers of chronic diseases						
0	Ref.					
1	1.16	<0.001	1.10-1.23			
>=2	1.37	<0.001	1.29-1.45			

Smoking status						
Never	Ref.			Ref.		
Previous	1.02	0.573	0.95-1.10	1.18	<0.001	1.09-1.28
Current	1.02	0.004	1.01-1.05	1.24	<0.001	1.15-1.35
Regular exercise						
Never	Ref.			Ref.		
Previous	1.02	0.572	0.94-1.12	0.90	0.034	0.88-0.96
Current	1.06	0.045	1.00-1.13	0.99	0.832	0.93-1.06
Dietary diversity score						
Poor	Ref.			Ref.		
Moderate	0.84	<0.001	0.80-0.89	1.03	0.387	0.97-1.10
Good	0.75	<0.001	0.69-0.80	0.94	0.048	0.86-0.98
Housework						
Almost everyday				Ref.		
Sometimes				0.97	0.440	0.88-1.06
Never				1.26	<0.001	1.17-1.35
Outdoor activities						
Almost everyday	Ref.			Ref.		
Sometimes	1.06	0.048	1.01-1.12	0.92	0.025	0.85-0.99
Never	1.04	0.262	0.97-1.12	0.97	0.528	0.89-1.06
Keeping pets or gardening						
Almost everyday	Ref.			Ref.		
Sometimes	0.84	0.003	0.75-0.94	0.92	0.023	0.86-0.99
Never	0.92	0.025	0.85-0.99	1.00	0.951	0.92-1.08
Reading books						
Almost everyday				Ref.		
Sometimes				1.24	0.001	1.09-1.41
Never				1.34	<0.001	1.21-1.49
Watching TV						
Almost everyday	Ref.			Ref.		
Sometimes	1.01	0.712	0.95-1.08	1.19	<0.001	1.10-1.28
Never	1.08	0.016	1.02-1.16	1.32	<0.001	1.23-1.42
Social participation						
Almost everyday				Ref.		
Sometimes				1.16	0.169	0.94-1.43
Never				1.57	<0.001	1.29-1.91
Activities of daily living						
Independent				Ref.		
Disabled				1.40	<0.001	1.30-1.51
Self-reported quality of life						
Good	Ref.			Ref.		
General	1.07	0.019	1.01-1.13	0.79	<0.001	0.74-0.84

Poor	1.29	<0.001	1.15-1.46	0.81	0.004	0.70-0.93
Self-reported health						
Good	Ref.			Ref.		
General	1.43	<0.001	1.35-1.51	0.88	<0.001	0.83-0.94
Poor	1.99	<0.001	1.84-2.15	1.21	<0.001	1.11-1.33

Notes: Sleep duration were divided by short (<7h), normal (7h-9h), and long (>9h) duration.
